# Establishment and Characterization of MCA23, a Novel Mouse Intrahepatic Cholangiocarcinoma Cell Line

**DOI:** 10.1002/cam4.71560

**Published:** 2026-01-29

**Authors:** Yuchao He, Yi Luo, Liwei Chen, Yu Wang, Bei Liu, Mengting Sun, Wenchen Gong, Xiangdong Tian, Lin Guo, Qin Zhang, Qiang Wu, Lu Chen, Hua Guo

**Affiliations:** ^1^ Department of Tumor Cell Biology Tianjin Medical University Cancer Institute and Hospital Tianjin China; ^2^ National Clinical Research Center for Cancer, State Key Laboratory of Druggability Evaluation and Systematic Translational Medicine, Tianjin Key Laboratory of Digestive Cancer, Tianjin's Clinical Research Center for Cancer Tianjin China; ^3^ Department of Pathology Tianjin Medical University Cancer Institute and Hospital Tianjin China; ^4^ Department of Endoscopy Tianjin Medical University Cancer Institute and Hospital Tianjin China; ^5^ Department of Genetics, School of Basic Medical Sciences Tianjin Medical University Tianjin China; ^6^ Department of Hepatobiliary Cancer, Liver Cancer Research Center Tianjin Medical University Cancer Institute and Hospital Tianjin China

**Keywords:** 3D models, cell line, chemoresistance, establishment, intrahepatic cholangiocarcinoma, metastasis

## Abstract

**Introduction:**

Intrahepatic cholangiocarcinoma (ICC) is an aggressive type of malignancy. Recent advancements have highlighted the importance of the tumor immune microenvironment in therapeutic responses and prognosis. However, the lack of a mouse‐derived ICC cell line and current mouse models limit explorations of the TME in ICC. Therefore, establishing suitable preclinical models is critical.

**Methods:**

In the present study, an ICC mouse model was established using hydrodynamic transfection. Primary ICC cells were isolated, purified, and cultured in the liver tissues of these mice. Cellular behaviors, molecular characterization, and genetic profiles were evaluated in vitro. The tumorigenic and metastatic potential of the cells was determined in vivo. Drug sensitivity was tested using organoids and micro‐dissected tumor tissues 3D models.

**Results:**

An ICC mouse model was successfully established based on pathological identification. Characterization confirmed that the MCA23 cell line was of ICC origin and maintained the morphological and molecular features of the primary tumor. The cells exhibited robust proliferative, migratory, and invasive capabilities, enabling the rapid formation of syngeneic tumors and metastases that were highly similar to the primary tumor. Genetic analysis revealed that the cell line was a new mouse‐derived cell line with cancer cell‐karyotype characteristics. Drug testing revealed varied responses to commonly used clinical chemotherapeutics for MCA23 tumors and metastases.

**Conclusion:**

MCA23 cell line provides a valuable experimental model for studying ICC pathogenesis, progression, metastasis, and drug‐resistance mechanisms. This model holds considerable promise for investigating the tumor immune microenvironment and potential immunotherapeutic approaches for advanced ICC.

**Abbreviations**
α‐SMA: alpha‐smooth muscle actin
AFP: alpha‐fetoprotein
Arg1: hepatic cell marker arginase 1
CK7: cytokeratin 7
CK19: cytokeratin 19
CTG: cell tracker green
EMT: epithelial‐mesenchymal transition
GPC3: glypican 3
H&E: hematoxylin and eosin
HTVi: hydrodynamic tail‐vein injection
ICC: intrahepatic cholangiocarcinoma
MDT: micro‐dissected tumor tissues
PI: propidium iodide
qRT‐PCR: quantitative real‐time polymerase chain reaction
STR: short tandem repeat
TEM: transmission electron microscopy
TME: tumor immune microenvironment
WB: Western blotting

## 1 Introduction

Intrahepatic cholangiocarcinoma (ICC) is the second most common primary liver tumor and a lethal malignancy in most patients [1]. The incidence of ICC has increased by more than 140% over the past four decades. Most patients have advanced‐stage disease at the first diagnosis. Even when detected early, small tumors may be situated in challenging locations within the liver and/or exhibit high levels of desmoplasia or paucicellularity, thereby limiting the sensitivity of cytological and pathological diagnostic methods [2]. Consequently, at the time of diagnosis, only 23%–53% of patients with ICC are eligible for surgical resection [3]. Even after curative‐intent surgical resection, the 5‐year overall survival rate ranges from approximately 20%–35% [4], with postoperative local and distant recurrences posing substantial obstacles to survival [5, 6]. Doublet chemotherapy with gemcitabine and cisplatin, along with targeted therapy and immunotherapy, often serves as the sole treatment option for patients with advanced disease. However, the effectiveness of these regimens is often hindered by inherent or acquired resistance of ICC to chemotherapeutic agents [1]. Recent advancements with triplet regimens and immunotherapy may herald a paradigm shift [1, 7, 8]. An increasing number of studies utilizing large‐scale genomics, transcriptomics, and epigenetics have partially revealed the comprehensive landscape and heterogeneity of ICC, further emphasizing the pivotal role of the immune microenvironment in treatment response and prognosis [9]. Thus, the development of appropriate experimental models in vitro and in vivo is essential for a thorough and detailed exploration of ICC, particularly the intricate interactions between tumor cells and the tumor immune microenvironment (TME).

Cell lines, which are utilized as in vitro models, play a significant role in a wide range of medical research fields, notably in fundamental tumor research and drug discovery. They offer a homogeneous source of biological material for experimental use [10]. Each cell line exhibits unique properties reflecting specific genotypes, sex‐related characteristics, and molecular phenotypes, making them highly valuable for scientific and commercial purposes [11]. Animal models play vital roles as transitional tools in the progression from cell lines to human clinical trials. These models serve as valuable resources for the study of carcinogenesis, tumor progression, and the assessment of the efficacy and toxicity of therapeutic compounds. In contrast to in vitro models, animal models offer a more accurate representation of the complex interactions between tumors and their microenvironment, support immune and vascular functions, clarify tumor cell mechanisms, and ultimately yield more clinically pertinent outcomes [12, 13]. However, the lack of mouse ICC cell lines has limited the use of ICC animal models. Most research on ICC models currently focuses on using human‐derived ICC cells in xenograft BALB/c nude mouse models [14, 15], which may not accurately represent the immune environment in patients or “real‐world” in the clinical setting. Additionally, the development of humanized immune system mouse models faces the challenge of high costs, thereby limiting their application. Therefore, the development of novel murine ICC cell lines is urgently required.

In the present study, we successfully established a novel and stable murine ICC cell line, MCA23. This cell line exhibited robust proliferative, migratory, and invasive properties, enabling the rapid formation of tumors and metastases in syngeneic immunocompetent C57/BL6 mice. Using organoid and microdissected tumor tissue (MDT) drug‐testing models, different responses to commonly used clinical chemotherapeutics were observed in MCA23 syngeneic tumor tissues and metastases. This cell line provides a valuable experimental model for studying the pathogenesis, progression, and dissemination of ICC. In particular, the TME, immunotherapy interventions, and drug resistance mechanisms were investigated.

## 2 Material and Methods

### 2.1 Hydrodynamic Injection and Establishment of Murine ICC Model

C57BL/6 mice (males, 6–8 weeks old) were purchased from Gempharmatech (Jiangsu, China) and housed in the SPF facility of the Tianjin Medical University Cancer Institute and Hospital. To establish a cholangiocarcinoma mouse model, hydrodynamic tail‐vein injection (HTVi) was adapted from previously established protocols [16]. Plasmids including pT3‐EF1α‐myrAKT, pT3‐EF1α‐YapS127A, and pCMV/SB11 were acquired from Addgene (Watertown, MA, USA). For each mouse, a solution containing 10 μg of pT3‐EF1α‐myrAKT, 10 μg of pT3‐EF1α‐myrAKT, and 5 μg of pCMV/SB11 was prepared in a 2.5 mL 0.9% NaCl saline. This plasmid mixture was then rapidly delivered to 8‐week‐old mice via HTVi, and the injection was completed within 5–7 s to ensure efficient plasmid delivery and expression. The entire procedure was conducted under strict aseptic conditions to minimize the risk of infection or contamination.

### 2.2 Establishment of Cell Line

Primary cells were isolated and cultured from freshly excised liver tissues of an ICC mouse model. Liver tissues were immediately rinsed in pre‐chilled DMEM/F12 medium supplemented 1% penicillin/streptomycin (catalog no. SV30010, HyClone, Logan, UT, USA) to eliminate blood contaminants. Non‐culturable tissues such as connective tissues were excised using surgical forceps. Tissue dissociation was performed using the Tumor Dissociation Kit (catalog no. 130–096‐730, Miltenyi, Germany). The cell suspension was filtered through 70 and 40 μm cell strainers (catalog no. 352,350 and 352,340, Falcon, USA) to remove any remaining tissue fragments. Red blood cells were lysed with ACK lysis buffer (catalog no. R1010, Solarbio Life Sciences, China), and the remaining cells were centrifuged and resuspended in the prepared primary cell medium with 10% FBS (catalog no. PWL040, Meiluncell, China) and cultured in a humidified 5% CO_2_ incubator at 37°C. Subsequent passagings and purification procedures were performed to establish the MCA23 cell line. Fibroblasts were removed by partial trypsinization. MCA23 cell line was cultured in Dulbecco's modified Eagle's medium (DMEM) (catalog no. C11995500BT, Gibco, USA) with 10% fetal bovine serum (FBS) (catalog no. ST30‐3302, PAN, Germany) and 1% penicillin/streptomycin (catalog no. SV30010, HyClone, USA) at 37°C, 5% CO_2_ in a humidified incubator.

Cells are planning to deposit in the China Center for Type Culture Collection (CCTCC) after publication.

### 2.3 Statistical Analysis

All data are presented as means ± standard error (SD) from at least three independent biological replicate experiments. Statistical analyses were conducted using GraphPad Prism 9.0, analysis of variance (ANOVA) or two‐tailed Student's *t*‐test as appropriate. Survival analyses were performed using the Kaplan–Meier method, and survival curves were compared using the log‐rank test. The significance levels were denoted as follows: *p < 0.05, **p < 0.01, ***p < 0.001, ****p < 0.0001, and n.s., not significant.

Other material and methods were presented in Doc. S1 of Supporting Information including hematoxylin and eosin (H&E), immunohistochemistry (IHC), immunofluorescence staining, morphological and transmission electron microscopy examinations, cell cycle detection by flow cytometry, cell viability assay and population‐doubling time analysis, colony formation assay, migration and invasion assays, western blotting and qRT‐PCR analyses, short tandem repeat (STR) detection, karyotypic analysis, subcutaneous syngeneic tumor model, organoid model, drug testing, micro‐dissected tumor tissues (MDT) model and drug testing assays.

## 3 Results

### 3.1 Establishment and Histological Characteristics of the Mouse Cholangiocarcinoma Model

**FIGURE 1:**
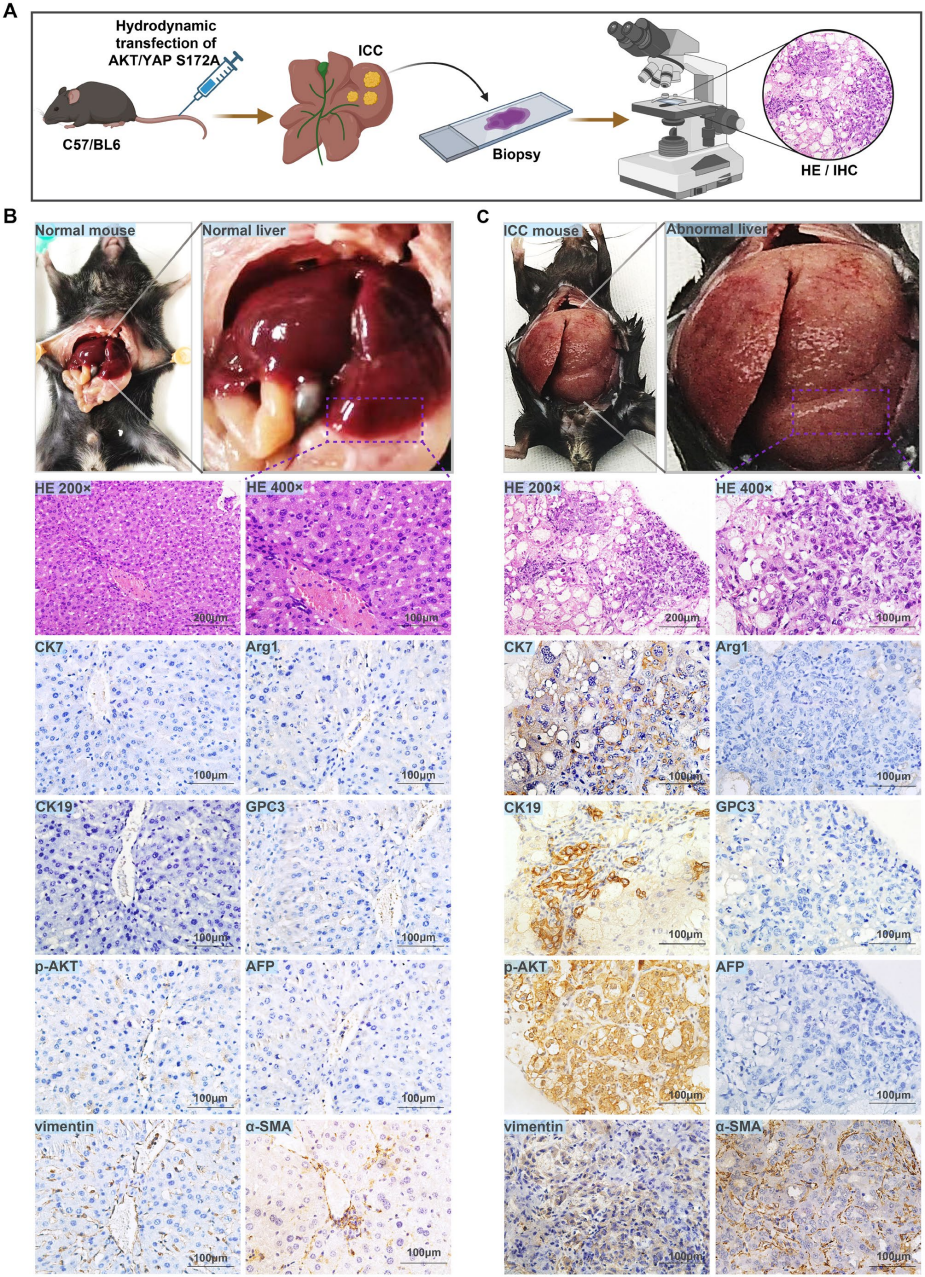
Establishment and histological features of the mouse model of cholangiocarcinoma. (A) Experimental workflow for ICC induction via hydrodynamic injection of AKT/YAP S127A plasmids. (B and C) Macroscopical and histological analyses of liver tissues from normal and ICC model mice, stained with H&E, CK7, CK19, Arg1, GPC3, AFP, p‐AKT, vimentin, and α‐SMA, to confirm ICC development.

Because of the elevated levels of AKT and YAP expression in human ICC [17], we administered plasmids encoding AKT/YAP S127A variants into the tail vein of C57BL/6 mice to induce ICC through hydrodynamic injection of sleeping beauty transposase. Subsequent biopsy and histological analyses confirmed the validity of the model (Figure 1A). Compared to normal mice, ICC model mice exhibited pronounced morphological abnormalities in the liver, such as irregular surfaces and nodular formations indicative of tumor presence, along with an increase in liver size and noticeable hardening of texture (Figure 1B,C). H&E staining revealed a disrupted architecture and ductular phenotypes that closely resembled those of human ICC. Additionally, histochemical staining demonstrated significantly positive expression of the cholangiocyte markers CK7 and CK19 [18, 19], whereas the hepatic cell markers Arg1, GPC3, and AFP [20] were negative (Figure 1B,C). Notably, the tumors showed marked activation of p‐AKT, distinct from normal liver tissue, and the tumor stromal cells stained positively for mesenchymal markers like vimentin and *α*‐SMA, underscoring the characteristic desmoplastic response seen in human ICC [17]. These findings, coupled with the surface morphology and histological characteristics of the liver, unequivocally established AKT/YapS127A tumors as ICC.

### 3.2 Establishment of Cell Line and Morphology Characteristics

**FIGURE 2:**
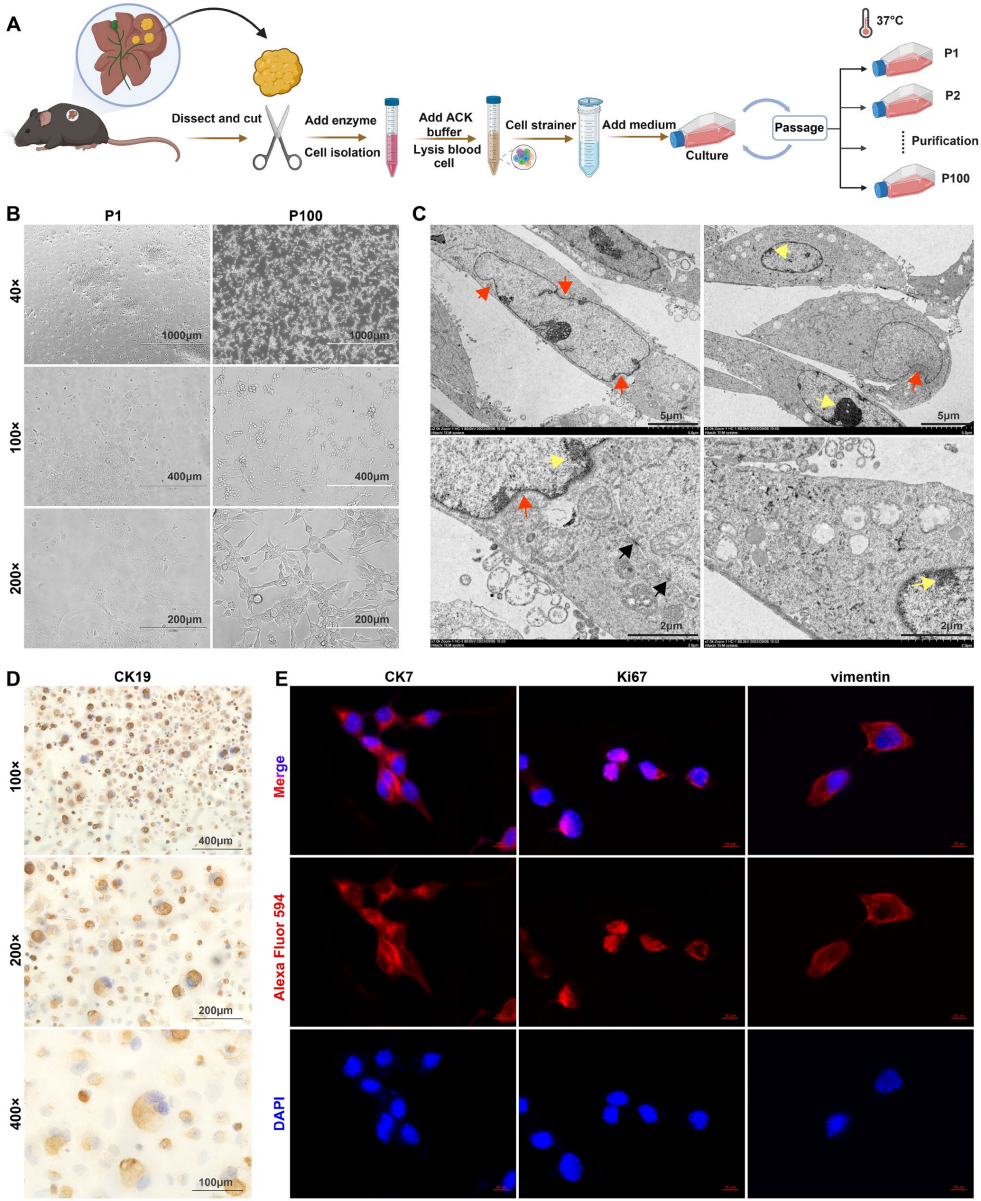
Establishment of cell line and morphology characteristics. (A) Experimental workflow for cell line generation. (B) Representative morphology of MCA23 cells at passages 1 and 100. Scale bar, 40×, 1000 μm; 100×, 400 μm; 200×, 200 μm. (C) Electron microscopy images of MCA23 at passage 100. Ultrastructural features (indicated by arrows) of MCA23 cells all demonstrated indented nuclear membranes (red arrows), hyperchromatic accumulation and margination (yellow arrows), and intercellular connections (black arrows). (D) IHC staining image of CK19 in MCA23 cell. Scale bar, 100×, 400 μm; 200×, 200 μm; 100×, 100 μm. (E) Representative image of CK7, Ki67, and vimentin by immunofluorescence staining in MCA23 cells. Scale bar, 10 μm.

Initially, a mouse model that had been previously identified with ICC was selected, and primary tumor tissues were extracted. Following physical fragmentation, enzymatic digestion, and elimination of residual tissue debris through filtration, a single‐cell suspension was collected for culturing. Through serial passaging and purification, a mouse ICC cell line named MCA23 was established, which was maintained through continuous passaging, as shown in the workflow in Figure 2A. The morphology of MCA23 cells was periodically observed under an inverted microscope during long‐term culture in vitro (> 100 passages). Following passaging and purification, the cells formed an adherent monolayer in a densely packed arrangement across the entire plate, suggesting loss of contact inhibition and increased proliferative capacity. Most of the cells displayed a spindle‐shaped morphology with consistent characteristics. The cells also exhibited sustained metabolic activity, with cell shape and growth rate remaining stable even after 100 passages (Figure 2B). Electron microscopy revealed that MCA23 cells had heterogeneous and irregularly shaped nuclei with irregular indentations in the nuclear membrane. It also contained many mitochondria and a rough endoplasmic reticulum. The chromatin distribution was also aberrant and characterized by hyperchromatic accumulation and margination (Figure 2C). Immunostaining demonstrated the expression of cholangiocyte markers CK19 (Figure 2D) and CK7 (Figure 2E), suggesting that the cell line originated from the biliary tract. Additionally, positive expression of the proliferation marker Ki67 and the mesenchymal marker vimentin was sustained even after 100 passages in the MCA23 cell line (Figure 2E). This finding indicated a consistent and stable cellular phenotype.

### 3.3 Cell Functional Properties: Proliferation, Migration, and Invasion Ability in Vitro

**FIGURE 3:**
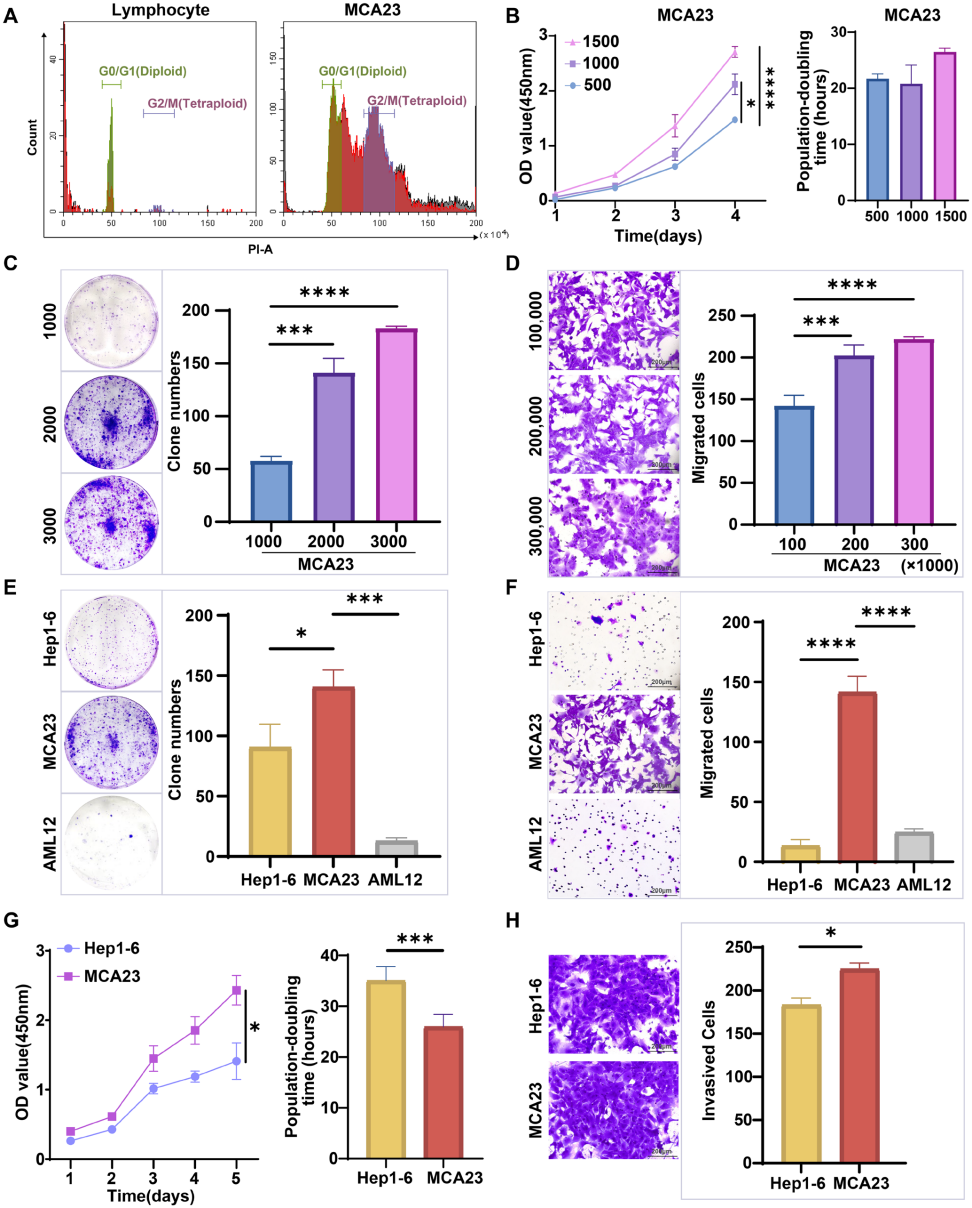
Functional properties of MCA23 cells. (A) DNA content and cell cycle distribution of normal lymphocytes and MCA23 cells. (B) Growth rate curves of MCA23 cells determined by CCK‐8 at a seeding density of 500, 1000, and 1500 cells. (C) Colony formation of MCA23 cells at a seeding density of 1000, 2000, and 3000 cells/well. (D) Migrated MCA23 cells through the transwell chamber after 8 h at different seeding densities. (E and F) Comparison of Hep1‐6, AML12, and MCA23 cells was conducted in terms of their colony formation at a seeding density of 2000 cells/well (E), and migration ability after 8 h at a seeding density of 10^5^ cells/well (F). (G and H) Proliferative capacity at a seeding density of 1000 cells/well (G) and invasive potential after 24 h at a seeding density of 10^5^ cells/well (H) were assessed in Hep1‐6 and MCA23 cells.

Cells in the G0/G1 phase were diploid, whereas those in the G2/M phase were tetraploid [15, 21]. DNA content was quantified by PI staining and flow cytometry. Unlike diploid lymphocytes, MCA23 cells exhibited a distinct polyploid phenotype (Figure 3A). Additionally, the proliferative characteristics of the MCA23 cell line were evaluated using CCK‐8 and colony formation assays. The results indicated that the proliferative capacity of MCA23 cells was concentration‐dependent and the growth rate increased with increasing cell‐seeding density. Specifically, the cells entered the logarithmic growth phase on the 2nd day post seeding, with a calculated population doubling time ranging from 21.69 to 26.47 h (Figure 3B). Notably, MCA23 cells showed the ability to form colonies when seeded with 1000, 2000, or 3000 cells, and their proliferative potential gradually increased as the number of plated cells increased (Figure 3C). Remarkably, even at a seeding density of 100,000 cells, MCA23 cells displayed significant migratory capability (Figure 3D). Furthermore, compared to normal hepatocyte AML12 and murine hepatocellular carcinoma (HCC) cell Hep1‐6, MCA23 cells exhibited significantly stronger clone‐forming ability (Figure 3E), migratory potential (Figure 3F), proliferative capacity (Figure 3G), and invasiveness (Figure 3H) than Hep1‐6 cells. In addition, we conducted additional experiments comparing MCA23 with the human ICC cell line RBE. Results demonstrated that MCA23 exhibits significantly enhanced proliferative and invasive capacities compared to RBE (*p* < 0.0001), while cisplatin sensitivity remained comparable between the two lines (Figure S2). These findings indicate that MCA23 cells exhibit typical malignant biological behavioral characteristics of cancer cells.

### 3.4 Molecular Properties and Multipotential Markers of MCA23 Cells

**FIGURE 4:**
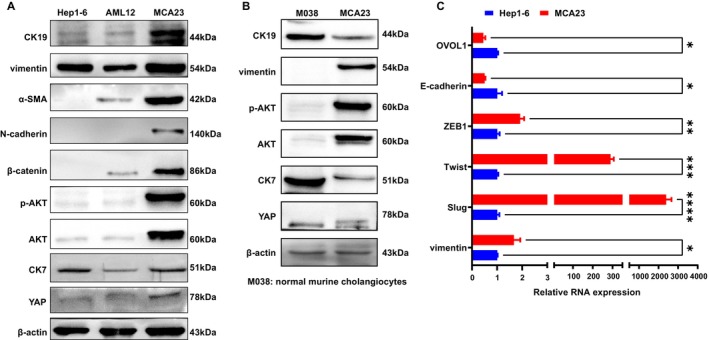
Molecular and biomarkers of MCA23 cells. (A) Western blot analysis comparing CK19, vimentin, N‐cadherin, α‐SMA, β‐catenin, p‐AKT, AKT, CK7, and YAP protein expression in Hep1‐6, AML12, and MCA23 cells. (B) Western blot analysis comparing CK19, vimentin, p‐AKT, AKT, CK7, and YAP protein expression in normal mouse intrahepatic bile duct epithelial cells (M038) and MCA23 cells. (C) RT‐PCR analysis comparing OVOL1, E‐cadherin, ZEB1, Twist, Slug, and vimentin relative RNA expression between Hep1‐6 and MCA23 cells.

Western blot and RT‐PCR analyses were used to characterize the molecular expression profiles and multipotent markers of the established MAC23 cell line. Compared to AML12 and Hep1‐6 cells, MAC23 cells exhibited a marked stronger expression of the biliary marker CK19 and mesenchymal markers (vimentin, N‐cadherin, and α‐SMA). Significantly, the upregulation of β‐catenin, a marker linked to unfavorable prognosis in cholangiocarcinoma [22], was observed in MCA23 cells. Furthermore, activation of p‐AKT and YAP, oncogenic events related to cholangiocarcinogenesis, was notably upregulated in MAC23 cells (Figure 4A). Direct comparisons of MCA23 and normal mouse intrahepatic bile duct epithelial cells (M038) showed that both express cholangiocyte markers CK7 and CK19; however, MCA23 exhibits significantly elevated AKT/p‐AKT and vimentin levels, which further supports its malignant cholangiocarcinoma phenotype (Figure 4B). qRT‐PCR analysis showed a significant upregulation of the epithelial–mesenchymal transition (EMT)‐related transcription factors ZEB1, Twist, and Slug [23], along with vimentin, in MCA23 cells compared to that in Hep1‐6 cells. Conversely, the expression levels of the mesenchymal‐epithelial transition‐related transcription factors OVOL1 [23] and E‐cadherin decreased in MCA23 cells, suggesting a heightened malignant metastatic propensity of MCA23 cells (Figure 4C).

### 3.5 Genetic Characteristics of MCA23 Cells

**FIGURE 5:**
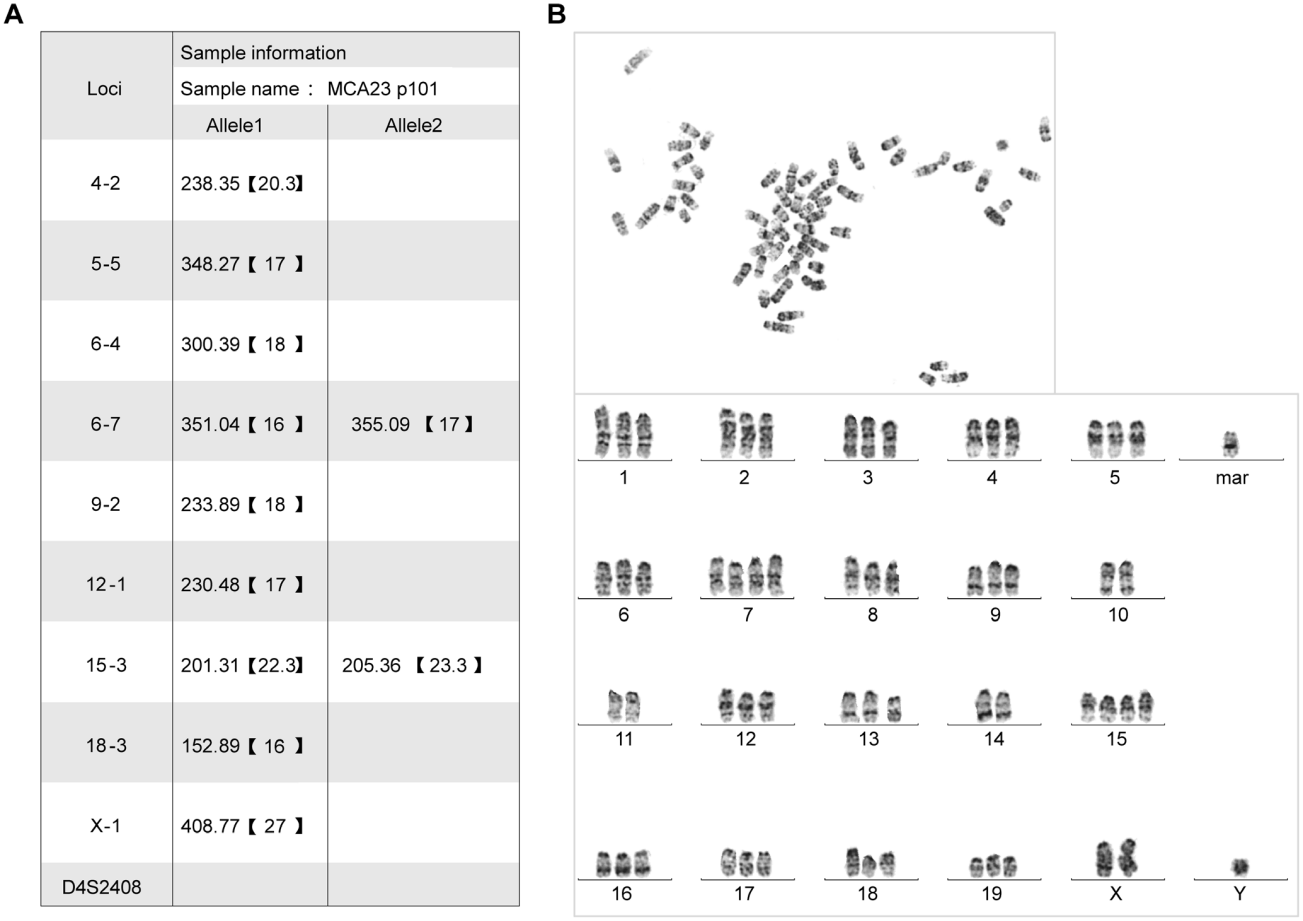
Genetic characteristics. (A) STR profile of MCA23 cells. (B) Karyotypic analysis showing representative G‐banding results and cytogenetic abnormalities.

DNA fingerprinting analysis of the 101st passage MCA23 cells revealed their murine origin, as evidenced by the absence of the human‐specific D4S2408 locus, thereby ruling out the possibility of human contamination. Furthermore, the distinct STR profile of this cell line did not match that of any of the existing cell lines in public databases, including ATCC, DSMZ, JCRB, ECACC, GNE, and RIKEN. This finding indicates the successful establishment of a novel murine‐derived ICC cell line (Figure 5A and Figure S1). Subsequent G‐banding chromosome karyotype analysis of MCA23 cells revealed a significant level of chromosomal heterogeneity, primarily manifesting as a triploid state. Notably, the presence of structural aberrations, particularly the distinctive mar1 marker, further characterized this cell line (Figure 5B). Such gross chromosomal abnormalities are often correlated with undefined cell growth potential, suggesting heightened malignancy in this novel murine‐derived ICC cell line.

### 3.6 Tumorigenic and Metastatic Properties of MCA23 Cells

**FIGURE 6:**
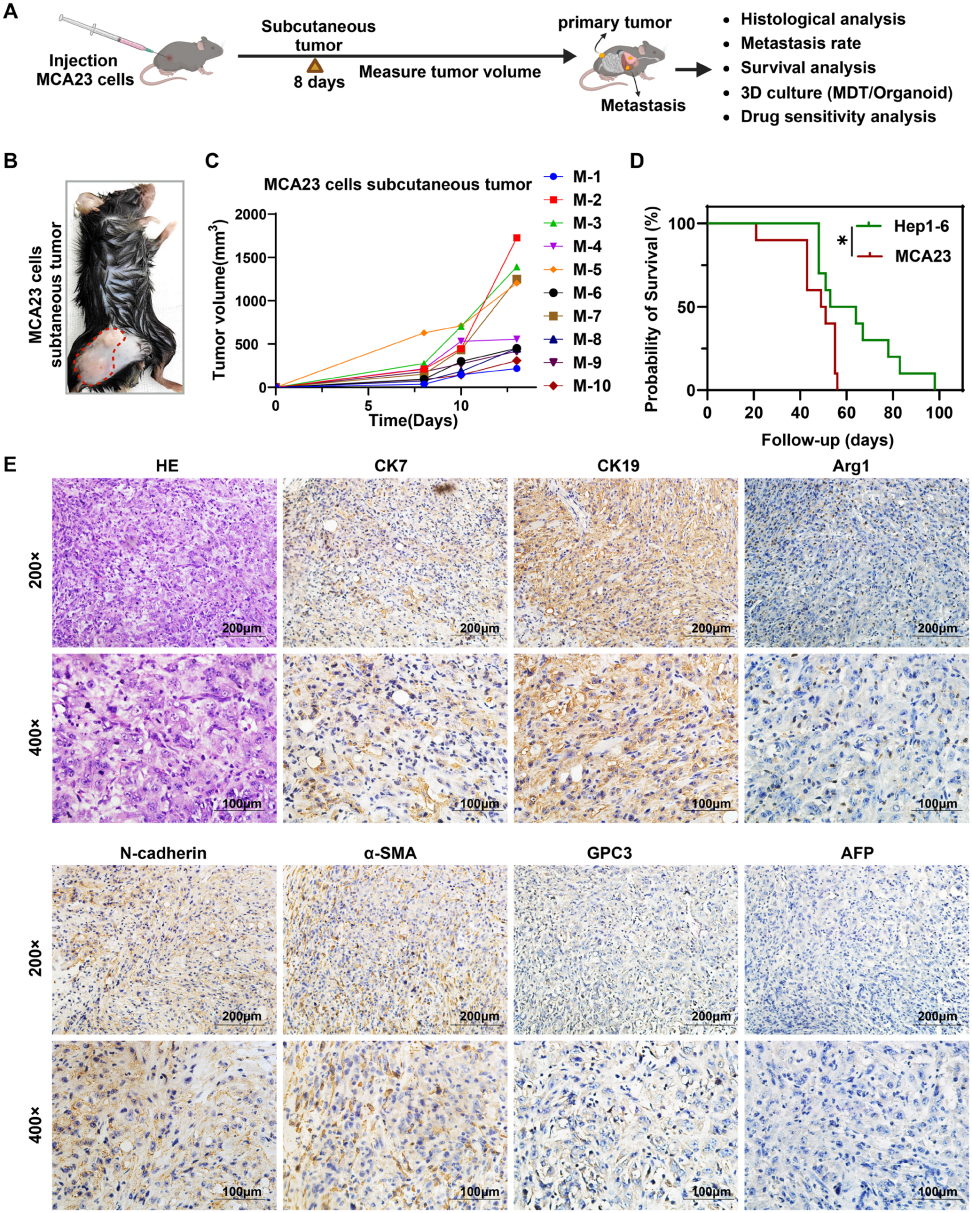
Tumorigenic properties. (A) Timeline and schedule of procedures in MCA23 subcutaneous tumor‐bearing model. MCA23 cells were subcutaneously injected into C57BL/6 mice, and tumor growth, metastasis rate and survival rate were monitored. Subcutaneous tumor and metastasis tissues were used for IHC identification and drug sensitivity testing using organoid and MDT models. (B) Representative image of subcutaneous tumor‐bearing mouse. (C) Tumor growth curve of MCA23 tumors at indicated time points in 10 mice. (D) Comparison of Kaplan–Meier survival curves in mice with Hep1‐6 and MCA23 subcutaneous tumors (*n* = 10). (E) Representative image of H&E and IHC staining of CK7, CK19, Arg1, GPC3, AFP, N‐cadherin, and α‐SMA in MCA23 subcutaneous tumor tissues.

To assess the tumorigenic potential and metastatic capability of MCA23 cells, as well as their impact on mouse survival and drug response, we established a syngeneic tumor‐transplantation model by subcutaneous injection of MCA23 cells (Figure 6A). Within a week post‐inoculation, rapid tumor growth was observed in all 10 C57/BL6 mice, presenting as palpable subcutaneous tumors with an average volume of 393 mm^3^, resulting in a 100% rate of tumor formation (Figure 6B,C). Furthermore, compared to Hep1‐6 hepatoma cells, mice inoculated with an equivalent number of MCA23 cells exhibited a relatively shorter survival time. This finding suggests a higher degree of malignancy and poorer prognosis associated with MCA23 (Figure 6D). H&E and IHC staining validated the subcutaneous tumor tissues as ICC, characterized by positive expression for CK7 and CK19, negative expression for Arg1, GPC3, and AFP, and positive expression for N‐cadherin and *α*‐SMA (Figure 6E).

**FIGURE 7:**
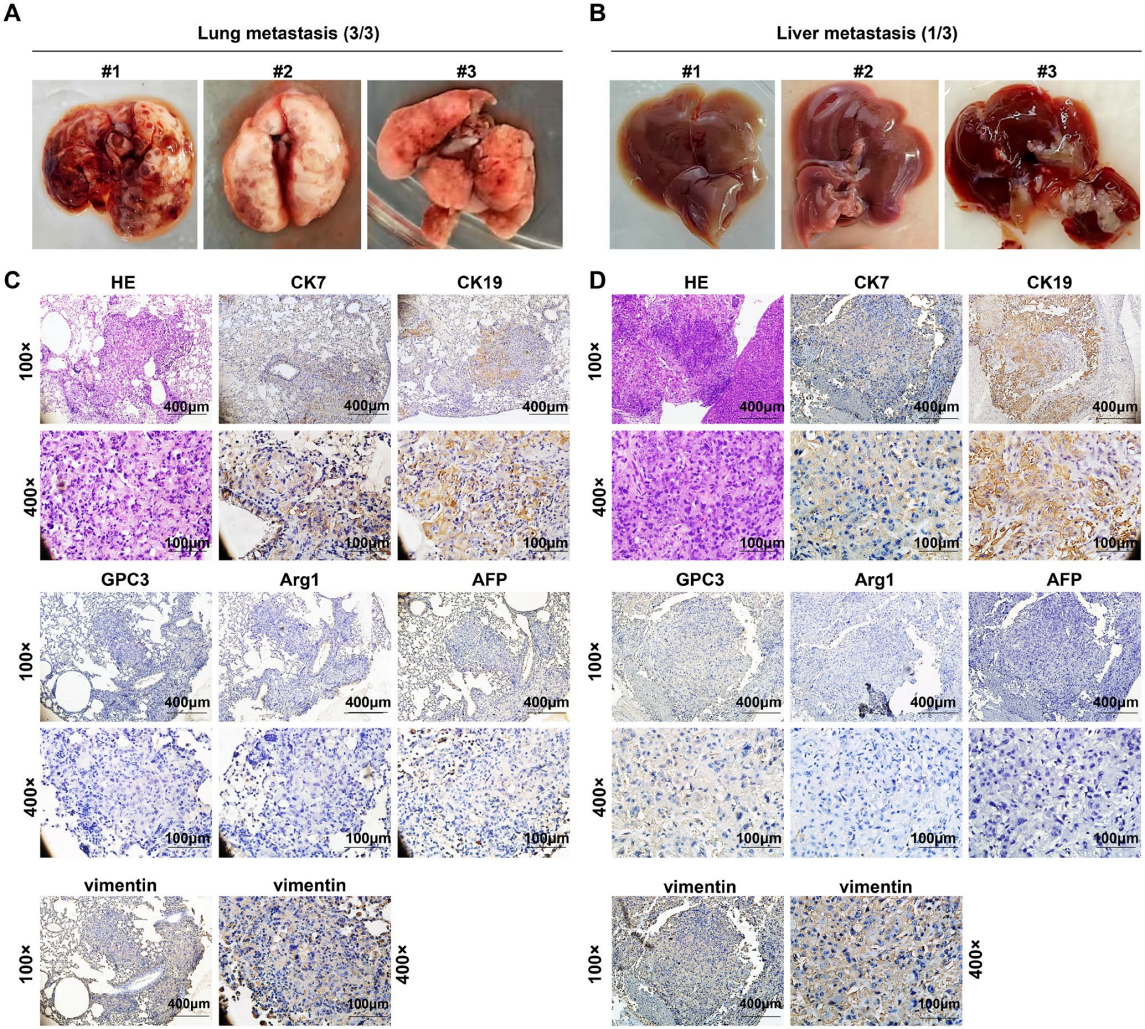
Metastatic properties. (A) Images of lungs with visible metastatic lesions in three tumor‐bearing mice. (B) Images of livers in three tumor‐bearing mice. (C,D) Representative image of H&E and IHC staining of CK7, CK19, Arg1, GPC3, AFP, vimentin, and α‐SMA in MCA23 lung (C) and liver (D) metastasis tissues.

We further examined the metastatic status of other organs in three additional tumor‐bearing mice. On day 40 post‐inoculation, all three mice exhibited visually detectable lung metastases (Figure 7A). Notably, one mouse (ID #3) presented with bloody ascites and abnormal liver morphology (Figure 7B). H&E and IHC staining confirmed 100% (3/3) lung metastasis rate and 33.33% (1/3) lung and liver metastasis rates, respectively. Metastatic lesions showed positive expression of CK7 and CK19 and negative staining for GPC3, Arg1, and AFP. Vimentin, which is associated with the promotion of EMT and metastasis, was positive (Figure 7C,D). This finding is consistent with their strong migratory and invasive abilities in vitro, indicating the significant tumorigenic and malignant metastatic potential in vivo for MCA23 cells.

### 3.7 The Tumor Immune Microenvironments in MCA23 Implanted Tumors

In addition, we have analyzed the tumor immune microenvironment of the MCA23 cholangiocarcinoma allograft model in C57BL/6 mice by utilizing flow cytometry assay at the experimental endpoint. The results demonstrate that in the MCA23 allograft tumors, in addition to tumor cells, various immune cell populations are present, including T/B cells, macrophages, neutrophils, and dendritic cells (DC) (Figure S3A). Additionally, we have provided flow cytometry data from three biological replicates, showing that CD45^+^ immune cells make up an average of 56.04% of the total live cell population in the tumor immune microenvironment (Mouse‐1: 43.89%, Mouse‐2: 60.66%, Mouse‐3: 63.56%) (Figure S3B). These findings suggest that our MCA23‐implanted syngeneic models hold significant potential for advancing research in cancer immunology.

### 3.8 Sensitivity to Chemotherapeutic Drugs

**FIGURE 8:**
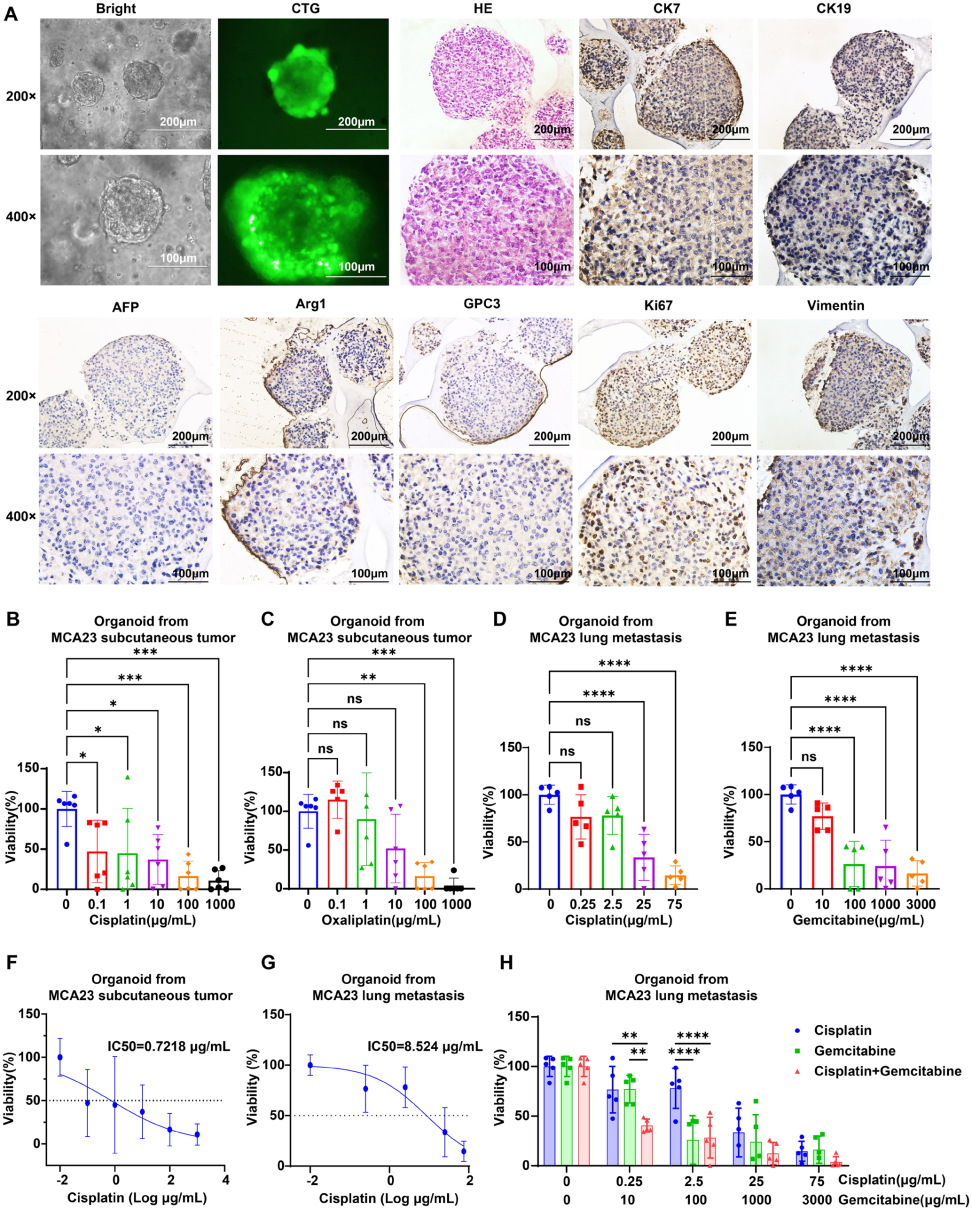
Organoid culture, identification, and drug sensitivity testing derived from MCA23 subcutaneous tumors and metastatic tissues. (A) Representative bright‐field microscope images of organoids, fluorescence images by CellTracker Green (CTG) labeling viable cells and identification images by H&E and IHC staining of CK7, CK19, Arg1, GPC3, AFP, vimentin, and α‐SMA in MCA23 tumor organoids. (B,C) Dose‐dependent effect of cisplatin (B) and oxaliplatin (C) in organoid from MCA23 tumor tissues as indicated treatment. (D,E) Dose‐dependent effect of cisplatin (D) and gemcitabine (E) in organoid from MCA23 metastasis tissues as indicated treatment. (F,G) Viability curves and IC50 values of cisplatin by CCK‐8 assay in organoid from MCA23 subcutaneous tumor (F) and metastasis tissues (G). (H) Effect comparison of cisplatin, gemcitabine monotherapy, and combined treatment in organoid from MCA23 metastasis tissues.

**FIGURE 9:**
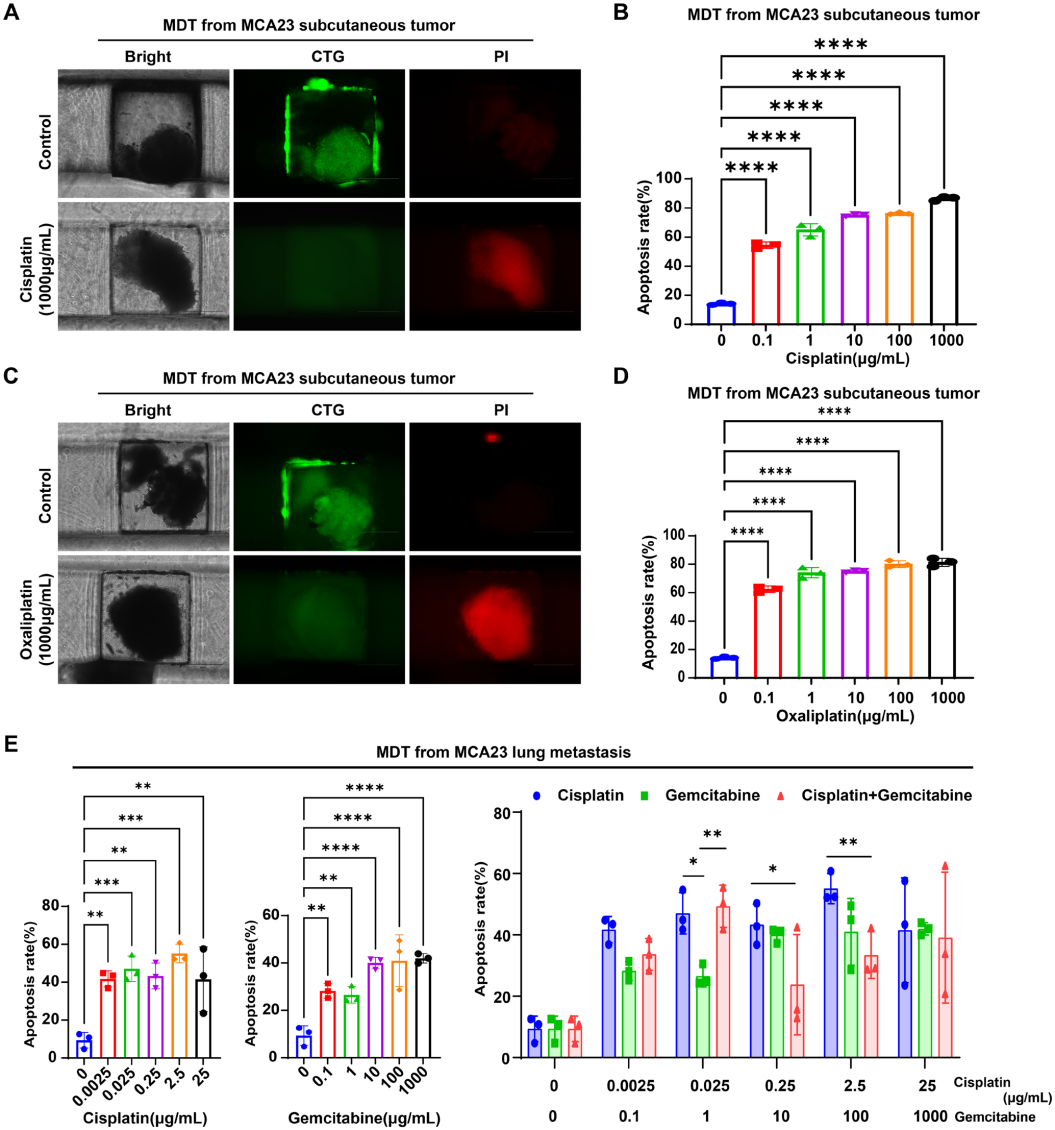
MDT culture, identification, and drug sensitivity testing derived from MCA23 subcutaneous tumors and metastatic tissues. (A) Representative bright‐field microscope images of MDTs from MCA23 subcutaneous tumor tissues, green fluorescence images by CellTracker Green (CTG) labeling viable cells, and red fluorescence images by PI labeling dead cells. (B) Apoptosis rates of MDTs from MCA23 subcutaneous tumor tissues detected by flow cytometry as indicated treatment of different dose of cisplatin. (C) Representative bright, CTG, and PI images of MDT from MCA23 metastasis tissues with or without treatment of oxaliplatin. (D) Apoptosis rates of MDTs from MCA23 metastasis tissues as indicated treatment of different dose of oxaliplatin. (E) Effect comparison of cisplatin, gemcitabine monotherapy, and combined treatment in MDTs from MCA23 metastasis tissues.

Cisplatin, oxaliplatin, and gemcitabine are chemotherapeutic agents frequently used for ICC treatment. The combination of gemcitabine and cisplatin is first‐line chemotherapy for unresectable ICC [24]. We utilized organoid models and MDTs‐on‐a‐chip, an ex vivo method of drug testing and personalized therapy [25], to evaluate the response of MCA23 subcutaneous tumors and lung metastases to these drugs. When cells from tumors and metastases were placed in Matrigel, the organoids grew from small granules to multiple spheroids of different sizes from 7 to 14 days (Figure 8A). The successful cultivation of organoids and their identification as ICC was confirmed using green fluorescence by CellTracker Green (CTG) labeling viable cells, the positive expression of CK7 and CK19, and the negative expression of Arg1, GPC3, and AFP. The presence of 50% positive Ki67 and vimentin expression indicated strong proliferative capacity and malignant potential (Figure 8A). Drug‐sensitivity testing revealed that organoids derived from MCA23 subcutaneous tumors exhibited relative sensitivity to cisplatin (IC50 = 0.7218 μg/mL) and oxaliplatin (IC50 = 7.3 ng/mL) (Figure 8B,C,F). However, organoids originating from MCA23 lung metastases displayed natural resistance to cisplatin (IC50 = 8.524 μg/mL) and gemcitabine (IC50 = 45.59 μg/mL) (Figure 8D,E,G), with no significant advantage observed when combining these drugs compared to monotherapy (Figure 8H). Consistently, the MDTs derived from MCA23 tumors were sensitive to cisplatin and oxaliplatin (Figure 9A–D) but resistant to cisplatin and gemcitabine in lung metastases. Combination therapy failed to show superior efficacy compared to monotherapy (Figure 9E).

## 4 Discussion

ICC is a highly invasive and lethal malignancy, with a high rate of metastasis. The heterogeneity of ICC at the multiomics and histopathological levels significantly hampers the efficacy of conventional treatments. To address this challenge, the integrated molecular landscape of ICC was revealed through multiomic sequencing, pointing to immune‐linked subtypes and drawing attention to the critical role of the TME in treatment response and prognosis [9, 12]. Appropriate animal models are critical for the advancement of new immunotherapies for ICC and for understanding immune–tumor interactions.

Current in vivo ICC models primarily use patient‐derived xenografts (PDX) and organoids in severely immunodeficient mice [26]. However, the success rates of PDX engraftment and organoids are relatively low, depending on the primary tumor and experimental design [26, 27]. Moreover, these models are effective for drug screening against tumor cells but lack the ability to study tumor‐immune interactions and drug effects on the immune system. They also do not allow the study of crosstalk among tumor cells, the multicellular microenvironment milieu, or the immune system [27, 28, 29, 30]. Humanized immune mice have the limitations of high cost and immune status variability; therefore, they are not ideal for studying the TME and drug effects in ICC [31]. Conversely, mouse syngeneic models allow for the injection of murine ICC cells into an immunocompetent host with a fully functional immune system, displaying significant advantages for studying the mechanisms of ICC and TME communication [26]. Our study successfully established an ICC murine model using HTVi as previously described [17], but this model led to diffuse growth of tumors in the liver and severe liver damage within 5 weeks. This limitation reduces the experimental window for drug research, making it difficult to explore the effects of drugs with long‐term dependence. Transgenic spontaneous tumor models may introduce bias by altering the expression of genes in non‐tumor cells.

In this study, the MCA23 murine ICC cell line was established using an ICC mouse model. It has been characterized and validated by analyzing its morphological, genetic, and pathological features. Even though the establishment of a syngeneic cholangiocarcinoma cell line and its use for allograft transplantation has also been demonstrated before in a hamster model [32, 33], the C57BL/6‐derived MCA23 cells exhibit differences in morphological and genetic profiles compared to hamster‐derived ICC cell lines (Ham‐1 and Ham‐2), suggesting divergent clonal origins and genomic instability profiles, suggesting MCA23 as a novel valuable resource for studying ICC heterogeneity. These tumors exhibit rapid growth, invasion, and metastasis. Implantation into immunocompetent mice successfully led to ICC lesions resembling human ICC. In addition, our immunocompetent C57BL/6 mouse model aligns more closely with human immunology [34, 35] and faces fewer limitations in experimental tool availability such as antibodies associated with immune cells for flow cytometric analysis. Our flow cytometry data quantitatively demonstrated the infiltration of diverse immune subsets (T/B cells, macrophages, DCs, neutrophils) in MCA23 tumors, offering a comprehensive characterization of the tumor immune microenvironment. This makes the MCA23 syngenetic C57BL/6 model overcome the limitations of existing models and provide an ideal experimental model for studying the interaction between the TME and tumors and for testing immunotherapy‐based strategies in ICC.

Furthermore, the increased invasive and metastatic capabilities of MCA23 compared to Hep1‐6 cells were coupled with elevated expression levels of EMT markers. Numerous studies have demonstrated a strong correlation between EMT and tumor metastasis [23, 36], which is consistent with our observation of high metastasis rates of MCA23 in vivo. Further characterization of this timeline in immunocompetent hosts in vivo, along with the isolation of tumor cells from the original injection and metastatic sites, may provide an excellent model for gaining valuable insights into the molecular mechanisms underlying ICC metastasis.

3D models are considered superior to 2D cultures for evaluating tumor characteristics. They hold great promise in predicting patient responses and bridging the gap between compound screening and clinical trials. Specifically, organoid models and MDTs on a chip are ex vivo 3D models of drug testing for personalized drug screening [25, 37]. Interestingly, we found that MDTs or organoids derived from MCA23 tumors in primary sites were relatively sensitive to cisplatin and oxaliplatin, but resistant to cisplatin and gemcitabine in lung metastasis sites, with the combination therapy failing to present superior efficacy compared to monotherapy. The different responses of primary and metastatic tumor tissues suggest clonal evolution and heterogeneity of ICC during recurrence and progression. Overall, the establishment of the MCA23 cell line contributes valuable data to preclinical research on ICC, opening new avenues for exploring the mechanisms of tumor metastasis and resistance, as well as for developing more personalized drug treatment strategies.

In conclusion, we have described the characteristics of an established cell line, MCA23, isolated from an ICC murine model using different techniques and approaches (morphology, phenotype, and genetics). Its use as a stable cell line, suitable for research on ICC pathogenesis, progression, metastasis, and drug resistance mechanisms, was confirmed through in vitro and in vivo studies. Importantly, the MCA23 model shows significant potential for exploring the TME and immunotherapeutic strategies for advanced ICC.

## Author Contributions

**Yuchao He:** conceptualization (equal), data curation (equal), formal analysis (equal), methodology (equal), project administration (equal), writing – original draft (lead), writing – review and editing (equal). **Yi Luo:** formal analysis (equal), methodology (equal), writing – original draft (supporting). **Liwei Chen:** formal analysis (equal), methodology (equal), writing – original draft (supporting). **Yu Wang:** data curation (supporting), visualization (supporting). **Bei Liu:** formal analysis (supporting), visualization (supporting). **Mengting Sun:** formal analysis (supporting), visualization (supporting). **Wenchen Gong:** data curation (supporting), visualization (supporting). **Xiangdong Tian:** data curation (supporting), visualization (supporting). **Lin Guo:** data curation (supporting), visualization (supporting). **Qin Zhang:** formal analysis (supporting), visualization (supporting). **Qiang Wu:** resources (supporting), supervision (equal), writing – review and editing (equal). **Lu Chen:** conceptualization (supporting), funding acquisition (equal), supervision (equal), writing – review and editing (equal). **Hua Guo:** conceptualization (lead), funding acquisition (lead), supervision (lead), writing – review and editing (lead).

## Funding

This study was supported by the National Natural Science Foundation of China (grants 82,173,208, 82,473,380, 82,103,672, 82,373,365), Tianjin Health Science and Technology Project (TJWJ2022MS008), Natural Science Foundation of Tianjin (24JCYBJC00700, 23JCYBJC00600), Scientific and Technological Projects of Tianjin (24ZXZSSS00050), and Tianjin Key Medical Discipline (Specialty) Construction Project (TJYXZDXK‐009A).

## Ethics Statement

All animal experiments in this study were conducted with the approval of the Animal Ethical and Welfare Committee of Tianjin Medical University Cancer Institute and Hospital (Tianjin, China), ensuring compliance with principles of animal protection, welfare, and ethics, as well as the relevant national regulations on animal welfare and ethics. The approval number is AE‐2021002 and the approval date is September 7, 2021. The animal ARRIVE guideline was followed when designing and performing animal experimentation [38].

## Consent

The authors have nothing to report.

## Conflicts of Interest

The authors declare no conflicts of interest.

## Supporting information


**Figure S1:** Electropherogram of MCA23 cell line.
**Figure S2:** The functional properties of MCA23 cells compared to other ICC cell lines. (A) Growth rate curves of MCA23 cells and RBE cells determined by CCK‐8 at seeding density of 1000 cells. (B) The invasive potential after 24 h at a seeding density of 105 cells/well was assessed in MCA23 and RBE cells. (C) Comparison of cisplatin sensitivity between MCA23 and RBE cells.
**Figure S3:** The tumor immune microenvironments in MCA23 implanted tumors. (A) Workflow of major immune cell panel in multiparameter flow cytometry analysis. (B) The representative flow cytometric plot of CD45+ immune cells in three MCA23 cholangiocarcinoma allograft models.

**Table S1:** Primary antibodies used in IHC, and immunofluorescence staining.

**Table S2:** Primary antibodies used in Western blotting.

**Table S3:** Primers used in qRT‐PCR.

**Data S1:** Supporting Information.

## Data Availability

All data and materials in the article are available upon reasonable request.
