# Supplementary material for: Establishment and Characterization of MCA23, a Novel Mouse Intrahepatic Cholangiocarcinoma Cell Line
Source: Cancer Med. 2026 Jan 29;15(2):e71560. doi: 10.1002/cam4.71560 (PMC12853219; doi:10.1002/cam4.71560)
Supplement: Supplementary file 1 — Figure S1: Electropherogram of MCA23 cell line. Figure S2: The functional properties of MCA23 cells compared to other ICC cell lines. (A) Growth rate curves of MCA23 cells and RBE cells determined by CCK‐8 at seeding density of 1000 cells. (B) The invasive potential after 24 h at a seeding density of 105 cells/well was assessed in MCA23 and RBE cells. (C) Comparison of cisplatin sensitivity between MCA23 and RBE cells. Figure S3: The tumor immune microenvironments in MCA23 implanted tumors. (A) Workflow of major immune cell panel in multiparameter flow cytometry analysis. (B) The representative flow cytometric plot of CD45+ immune cells in three MCA23 cholangiocarcinoma allograft models. [file CAM4-15-e71560-s005.docx]

**Supplementary figure 1**

**
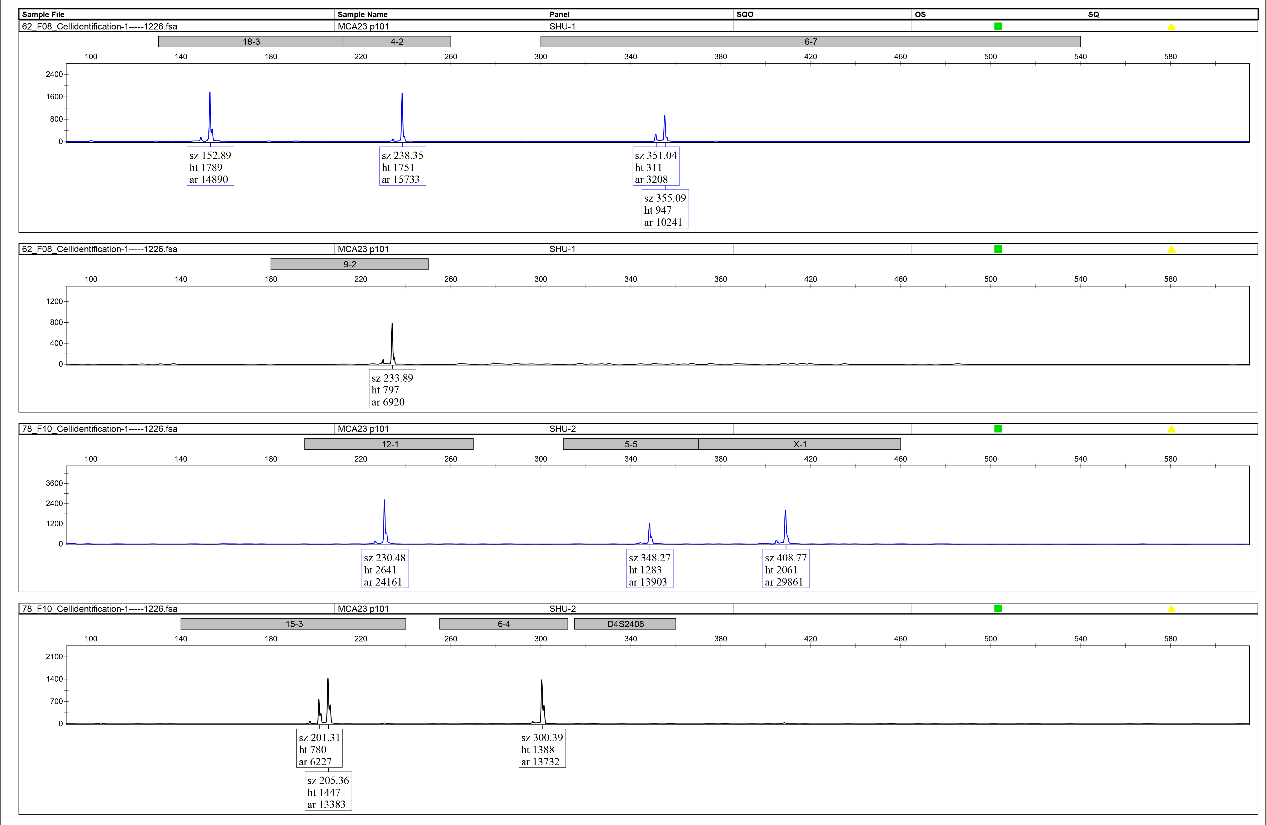
**

**Fig. S1 Electropherogram of MCA23 cell line.**


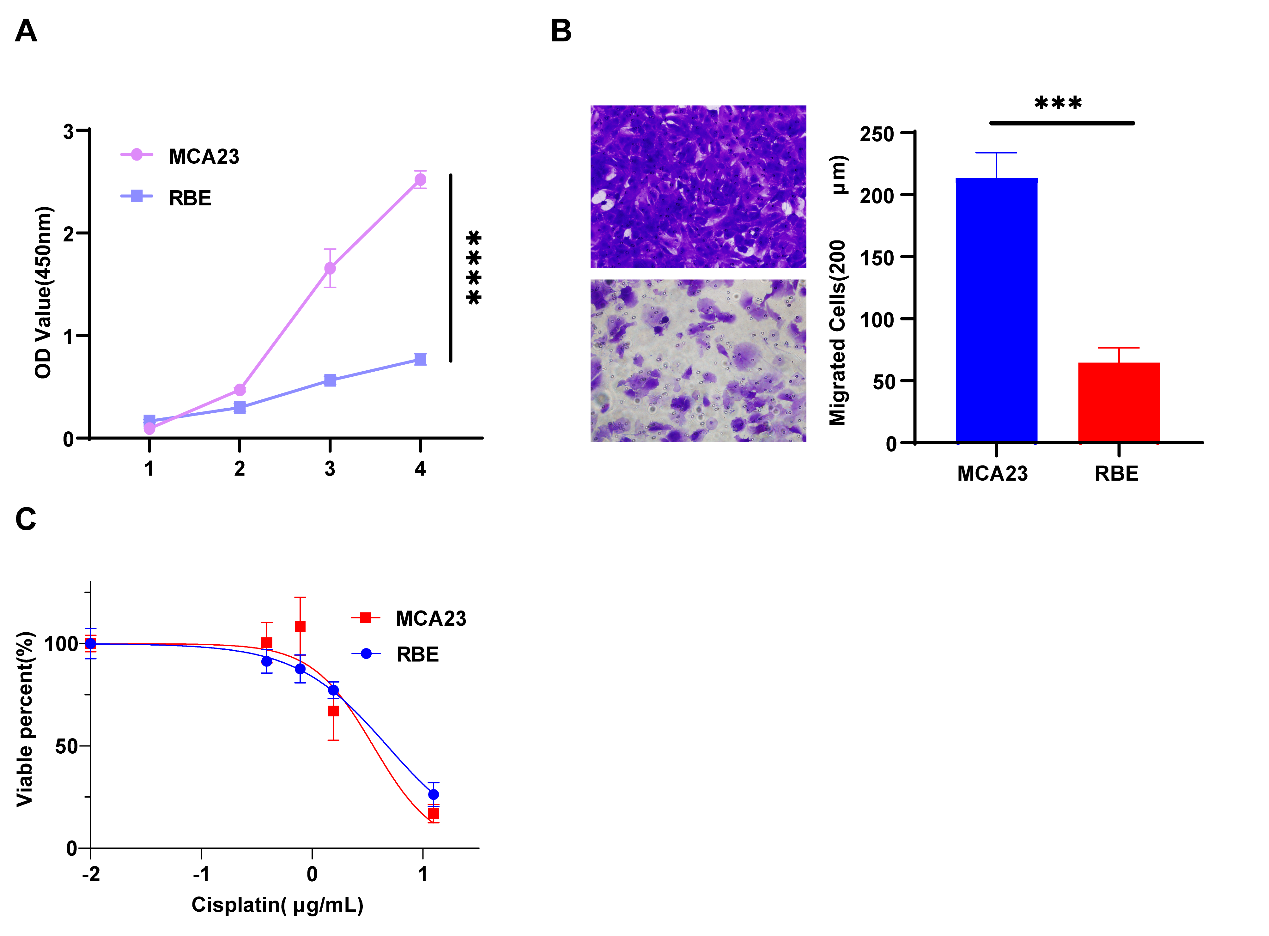


**Figure S2. The functional properties of MCA23 cells compared to other ICC cell lines. (A)** Growth rate curves of MCA23 cells and RBE cells determined by CCK-8 at seeding density of 1000 cells. **(B)** The invasive potential after 24 h at a seeding density of 10^5^ cells/well was assessed in MCA23 and RBE cells. **(C)** Comparison of cisplatin sensitivity between MCA23 and RBE cells.


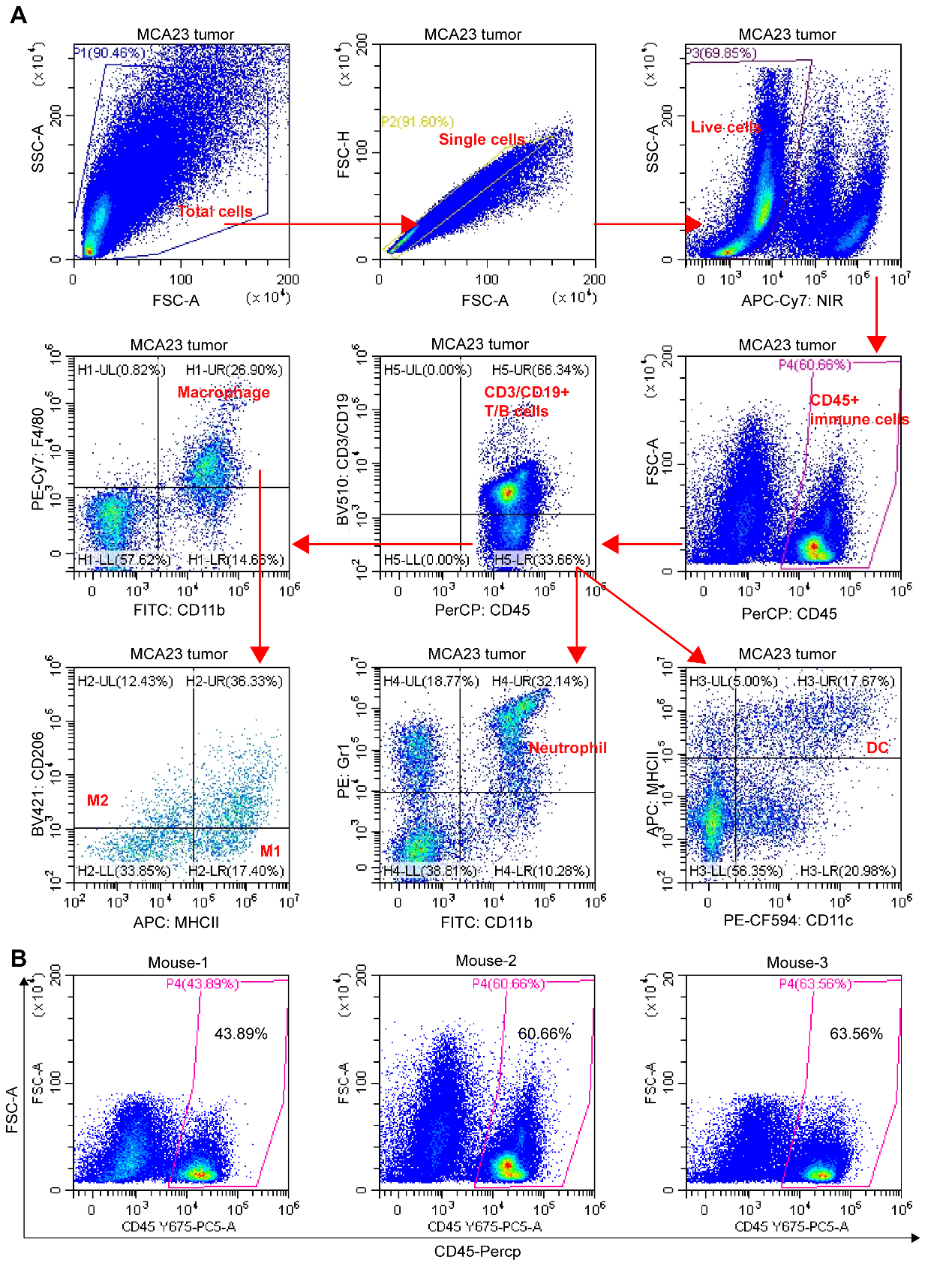
 **Figure S3. The tumor immune microenvironments in MCA23 implanted tumors.** (**A**) Workflow of major immune cell panel in multiparameter flow cytometry analysis. (**B**) The representative flow cytometric plot of CD45^+^ immune cells in three MCA23 cholangiocarcinoma allograft models.
