# Supplementary material for: Establishment and Characterization of MCA23, a Novel Mouse Intrahepatic Cholangiocarcinoma Cell Line
Source: Cancer Med. 2026 Jan 29;15(2):e71560. doi: 10.1002/cam4.71560 (PMC12853219; doi:10.1002/cam4.71560)
Supplement: Supplementary file 2 — Table S1: Primary antibodies used in IHC, and immunofluorescence staining. [file CAM4-15-e71560-s004.docx]

**Supplementary Table**

**Table S1** Primary antibodies used in IHC, and immunofluorescence staining

| **Antibody** | **Source** | **Catalog number** | **Dilution** |
| --- | --- | --- | --- |
| CK7 | Abclonal | #12004 | 1:100 |
| CK19 | Abmart | #T55312S | 1:100 |
| Arg1 | Abclonal | #A22410 | 1:100 |
| GPC3 | Abcam | #ab66596 | 1:50 |
| AFP | Proteintech | #14550-1-AP | 1:50 |
| Ki67 | Abclonal | #A16919 | 1:100 |
| vimentin | Abcam | #92547 | 1:250 |
| α-SMA | Abcam | #ab124964 | 1:400 |
