# Supplementary material for: Establishment and Characterization of MCA23, a Novel Mouse Intrahepatic Cholangiocarcinoma Cell Line
Source: Cancer Med. 2026 Jan 29;15(2):e71560. doi: 10.1002/cam4.71560 (PMC12853219; doi:10.1002/cam4.71560)
Supplement: Supplementary file 3 — Table S2: Primary antibodies used in Western blotting. [file CAM4-15-e71560-s003.docx]

**Supplementary Table**

**Table S2** Primary antibodies used in Western blotting

| **Antibody** | **Source** | **Catalog number** | **Dilution** |
| --- | --- | --- | --- |
| CK19 | ABclonal | # A0247 | 1:1000 |
| vimentin | Abcam | #92547 | 1:1000 |
| AKT | Cell Signaling Technology | #9272s | 1:1000 |
| p-AKT | Cell Signaling Technology | #s473 | 1:1000 |
| β-catenin | Abcam | # E247 | 1:1000 |
| N-cadherin | Cell Signaling Technology | #13116s | 1:1000 |
| α-SMA | Abcam | #ab124964 | 1:1000 |
| β-actin | Santa Cruz Biotechnology | #sc-47778 | 1:1000 |
