# Supplementary material for: Establishment and Characterization of MCA23, a Novel Mouse Intrahepatic Cholangiocarcinoma Cell Line
Source: Cancer Med. 2026 Jan 29;15(2):e71560. doi: 10.1002/cam4.71560 (PMC12853219; doi:10.1002/cam4.71560)
Supplement: Supplementary file 4 — Table S3: Primers used in qRT‐PCR. [file CAM4-15-e71560-s001.docx]

**Supplementary Table**

**Table S3** Primers used in qRT-PCR

| Primer | Sequence (5’ to 3’) |
| --- | --- |
| β-actin-Forward | CACTGTCGAGTCGCGTCC |
| β-actin-Reverse | CGCAGCGATATCGTCATCCA |
| OVOL1-Forward | GCTAAAGGACGCTGGCTTAG |
| OVOL1-Reverse | GTCTCGAAGGCTCATGTCCA |
| E-cadherin-Forward | AACCCAAGCACGTATCAGGG |
| E-cadhrerin-Reverse | ACTGCTGGTCAGGATCGTTG |
| ZEB1-Forward | GCGGCGCAATAACGTTACAA |
| ZEB1-Reverse | CTCGTTCTTCTCATGGCGGT |
| Twist-Forward | CGGCCAGGTACATCGACTTC |
| Twist-Reverse | GGGGGACACAAACGAGTGTI |
| Slug-Forward | GCCTCCAAGAAGCCCAACTA |
| Slug-Reverse | GCCGACGATGTCCATACAGT |
| vimentin-Forward | GGCTGCGAGAGAAATTGCAG |
| vimentin-Reverse | AGGCTTGGAAACGTCCACAT |
