# Supplementary material for: Establishment and Characterization of MCA23, a Novel Mouse Intrahepatic Cholangiocarcinoma Cell Line
Source: Cancer Med. 2026 Jan 29;15(2):e71560. doi: 10.1002/cam4.71560 (PMC12853219; doi:10.1002/cam4.71560)
Supplement: Supplementary file 5 — Data S1: Supporting Information. [file CAM4-15-e71560-s002.docx]

**Document S1**

**Supplementary material and methods**

**Hematoxylin and eosin (H&E)****, immunohistochemistry (IHC), and immunofluorescence staining**

H&E assay was performed as described in a previous study ^17^. Briefly, after establishing the model, the mouse tumor tissue was promptly fixed in a 4% formalin solution for 24 h, maintained at 4℃, dehydrated using ethanol, and embedded in paraffin. The samples were sectioned into uniform slices measuring 4 µm in thickness, mounted on glass slides, and subsequently stained with hematoxylin and eosin (H&E) (catalog no. G1120, Solarbio, China). The morphology of H&E saining was observed under the microscope.

IHC and immunofluorescence staining were applied to assess the protein levels of cytokeratin 7 (CK7), cytokeratin 19 (CK19), arginase 1 (Arg1), Glypican 3 (GPC3), alpha-fetoprotein (AFP), vimentin, alpha-smooth muscle actin (α-SMA), and Ki67 in the paraffin-embedded samples according to previously described methods ^18^.

Briefly, for IHC assay, Tissues were dewaxed, rehydrated through xylene and gradient ethanol, then subjected to pressurized antigen retrieval using citrate buffer (catalog no. ZLI-9065, ZSGB-BIO, China) for 2.5 min. Endogenous peroxidase activity was blocked with 3% H₂O₂. Primary antibody incubation occurred at room temperature (30 min) followed by overnight incubation at 4℃. After PBS washing, secondary antibody staining proceeded at 37℃ for 1 h. Visualization used DAB solution (catalog no. ZLI-9017, ZSGB-Bio, China) with hematoxylin counterstaining. The primary antibodies used in IHC staining are shown as Table S1.

For immunofluorescence staining, the slices were permeated with 0.2% TritonX-100 (catalog no. IT9100, Solarbio, China) for 30 min. 3% bovine serum albumin was then closed at room temperature for 30 min, then incubated with specific primary antibody described above at 4℃ overnight, incubated with fluorescent secondary antibody for 1 h in a wet box. After the addition of DAPI (catalog no. S2110, Solarbio, China) in the dark. The tablets were re-dyed for 5 min, and finally the sides were sealed with anti-fluorescent quench agent. The images were viewed using a fluorescence microscope.

**Cell culture**

Human ICC cell line RBE was purchased from Feiouer Biotechnology Co., Ltd. The cells were cultured in RPMI 1640 medium (Corning, NY, USA) supplemented with 10% fetal bovine serum (FBS, PAN–Seratech) and 1% penicillin/streptomycin (Hyclone). Hepa1-6 and AML12 cells were purchased from the American Type Culture Collection (Manassas, VA, USA). Normal mouse intrahepatic bile duct epithelial cells (M038) were purchased from Wuhan Procell Life Science and Technology Co., Ltd. (Wuhan, China). These cells were routinely cultured in DMEM (Corning, NY, USA) supplemented with 1% penicillin/streptomycin and 10% FBS in a humidified incubator at 37 °C with 5% CO_2_.

**Morphological and transmission electron microscopy examinations**

The established cells were routinely observed for morphology using a phase-contrast microscope. Ultrastructural characteristics were studied by transmission electron microscopy. The cells were fixed in 2.5% glutaraldehyde in phosphate buffer. Subsequently, the specimens were washed with phosphate buffer and fixed in % OsO_4_ in phosphate buffer for 1 h. After dehydration in graded ethanol solutions, the specimens were embedded in epoxy resin. Ultrathin sections were observed using a transmission electron microscope.

**Cell-cycle detection by flow cytometry**

Lymphocyte and MCA23 cells were digested and washed once with PBS. Then, cell suspensions in 50 μL of PBS were added to 10 mL 95% ethanol and maintained at 4 °C overnight. The next day, the cells were stained with 500 μL of propidium iodide (PI; catalog no. 550825, BD Biosciences, USA) after centrifugation and incubated in darkness for more than 30 min. The samples were analyzed on a ﬂow cytometer (CytoFLEX LX, Beckman, USA).

**Cell viability assay and population-doubling time analysis**

The MCA23 cell line was divided into 500, 1000, and 1500 cells on 96-well plates. To compare the proliferation ability of Hep1-6 and MCA23 cell line, the cells were plated onto a 96-well plate at 1000 cells/well. Then, we added 10 μL of CCK-8 reagent (catalog no. BS350B, Biosharp Life Sciences, China) in each well and co-incubated them with the cells in a 37 °C incubator for 4 h. The OD value at 450 nm was measured, and cell proliferation was monitored for 4–5 days. The measured OD value was used to convert the actual number of cells. The population-doubling time was obtained by entering the values into doubling time software (http://www.doubling-time.com).

**Colony formation assay**

For colony formation assay, the MCA23 cell line was divided into 1000, 2000, and 3000 cells on six-well plates. The MCA23, AML12, and Hep1-6 cell lines were plated onto a six-well plate at 2000 cells/well. After a week of incubation, the surviving colonies were fixed, stained with 0.5% crystal violet (catalog no. G1062, Solarbio Life Sciences, China), imaged for 30 min, and counted. The data were presented as the mean ± SD of triplicate dishes in the same experiment.

**Migration and invasion assays**

Cell suspensions containing 1×10^5^ cells in DMEM-only medium were plated onto the upper space of an 8-μm chamber (catalog no. 353097, Falcon, Corning, USA) with or without diluted Matrigel (catalog no. 356234, BD Biocoat, Corning, USA). The lower chamber holding a 24-well plate was filled with DMEM containing 10% FBS. After incubation for approximately 8 h for migration assay (without diluted Matrigel) or 24 h for invasion assay (with 50 μL of Matrigel diluted in PBS (1:3 ratio)) in a 37 °C incubator, the contents were cleaned with PBS and the chamber membrane was fixed and rinsed before being stained. Random fields were captured with an optical microscope for cell quantification. All measurements were detected in triplicate.

**Western blotting and qRT-PCR analyses**

Western blotting was applied to assess the protein levels of CK19, vimentin, N-cadherin, α-SMA, β-catenin, p-AKT and AKT in Hep1-6, AML12 and MCA23 cell lysates according to previously described methods ^18^. Briefly, the cells were washed thrice with PBS, followed by lysis using SDS lysis buffer on ice for 30 min. Subsequently, the protein samples were loaded into SDS-PAGE gel wells and separated into bands at a voltage range of 60 V to 120 V. The proteins were then transferred to a polyvinylidene fluoride membrane by electroblotting to facilitate interaction with antibodies. To prevent nonspecific binding, the membrane was blocked with 5% bovine serum albumin. The primary antibody was incubated with the membrane overnight at 4 °C, followed by incubation with the secondary antibody at room temperature for 1 h. Detection was carried out using ECL chemiluminescence. The primary antibodies used in Western blotting are shown in Table S2.

TRlzol reagent (catalog no. 15596018, Invitrogen, USA) was used for the extraction of total RNA from adherent cells in accordance with the manufacturer's guidelines. RNA was quantified with a a NanoDrop spectrophotometer (NanoDrop Technologies). Subsequently, cDNA was synthesized using the PrimeScript^TM^ RT Master Mix (catalog no. RR036A, TaKaRa, Japan). Quantitative RT-PCR (qRT-PCR) was performed using AceQ SYBR qPCR Master Mix (catalog no. Q111, Vazyme, China) according to the provided instructions. The specific primers utilized are detailed in Table S3.

**Short tandem repeat (STR) detection**

A total of 101 passages of logarithmic growth phase MCA23 cells were utilized in the experiments, with STR analysis conducted in accordance with the protocols outlined by the American National Standard Institute (ANSI/ATCC ASN-0002-2011 Authentication of Human Cell Lines: Standardization of STR Profiling). Briefly, DNA was extracted from MCA23 cells using TIANamp Genomic DNA Kits (Tiangen, China). The total amount of DNA was quantified using a NanoDrop 8000 spectrophotometer (Thermo Fisher Scientific). The nine mouse STRs including one human STR were amplified by multiplex PCR and separated on ABI 3730XL Genetic Analyzer. The signals were then analyzed by the software GeneMapper. The STR profiles were compared with those recorded in public cell banks (ATCC, DSMZ, JCRB, ECACC, GNE, and RIKEN) for reference matching with a standard match threshold of 80% according to the Tanabe formula.

**Karyotypic analysis**

Chromosomal analysis was conducted on cells at 101 passages through a series of standardized procedures. Specifically, the cells were exposed to 0.1 µg/mL colchicine for 1 h, followed by digestion with trypsin/EDTA and preparation of slides through established techniques. Hypotonic treatment was administered using a 0.075 M potassium chloride solution for 20 min at ambient temperature. The fixed cells on the slides were subsequently stained with trypsin-Giemsa to facilitate the identification of individual metaphase chromosomes. The chromosome karyotypes were analyzed using G.banding. Chromosome aberrations analysis was conducted using a Zeiss Axioscope inverted microscope and IKAROS software (Metasystems, Germany). Two maps were created, with 29 and 17 cells counted, showing chromosome numbers ranging within 27–121 and triploid levels.

**Subcutaneous syngeneic tumor model**

C57BL/6 mice (males, 4–8 weeks old) were purchased from Gempharmatech (Jiangsu, China) and housed in the SPF facility of Tianjin Medical University Cancer Institute and Hospital. MCA23 cells (1×10^6^ cells/mouse) were subcutaneously injected into the armpit of C57BL/6 mice (n=10). The mice were observed, and their tumor sizes were measured daily with a caliper after receiving the injection. The mice were sacrificed either at the end of the experiment or before the maximum tumor diameter reached 20 mm. To compare survival time after implanting MCA23 and Hep1-6 cells, 1×10^6^ cells of each type were injected into mice (n=10). Tumor volume = (length × width^2^)/2. In fact, when the maximum tumor length reached 15 mm, the corresponding tumor volume was 1727.81 mm^3^ at most, and the average tumor volume for the 10 mice was 797.4 mm^3^. At this point, a humane endpoint was performed, and the experiment was terminated in full compliance with ethical guidelines to prevent any undue distress to the animals. Final survival time was recorded until mice died. Metastasis was assessed by injecting 1×10^6^ MCA23 cells into three additional mice, which were sacrificed around day 50 to check for metastasis in the liver and lungs. Liver and lung metastases were achieved and identified by H&E and IHC staining. The study was approved by the Research Ethics Committee of the Tianjin Medical University Cancer Institute and Hospital in China (approve no. AE-2021002; approve date. September 7, 2021), and the study was conducted in compliance with the animal ARRIVE guideline.

**Flow cytometric assay for tumor immune microenvironment**

Tumor tissues were separated and cut into small pieces, digested for 1 hour at 37°C using the Tumor Dissociation Kit (catalog no. 130-096-730, Miltenyi, Germany). Spleens were ground in copper mesh, and the cells were filtered through 40 and 70 μm strainers (catalog no. 352340 and 352350, Falcon, Corning, USA). Red blood cells were lysed with ACK lysis buffer (catalog no. R1010, Solarbio Life Sciences, China) on ice for 5 minutes. 1×10^5^ cells were stained in 1.5-mL tubes with antibodies for 15 minutes at room temperature in the dark. The following antibodies were used: PerCP anti-mouse CD45 and PE anti-mouse Ly-6G and Ly-6C (Gr1) (catalog no. 552848 and 553128, BD Biosciences, Germany); PE-Cy7 anti-mouse F4/80 (catalog no. 25-4801-82, eBioscience, USA); Brilliant Violet 510 anti-mouse CD3/CD19, Brilliant Violet 421™ anti-mouse CD206, APC anti-mouse I-A/I-E (MHCII), PE/Dazzle™ 594 anti-mouse CD11c, and Zombie NIR Fixable Viability (catalog no. 100234/115546, 141717, 107614, 117348 and 423105, Biolegend, USA); FITC anti-mouse CD11b (catalog no. FHF011b-02-100, Sizhengbai Biotechnology, China). After incubation, 400 μL of PBS was added to each tube to wash the cells. The samples were then analyzed using a FACS Aria flow cytometer (BD) with CellQuest software, and the data were analyzed using FlowJo software.

**Organoid model and drug testing**

In a typical procedure, 1000–1500 mm^3^ large tumor tissues were resected and dissociated using scissors, followed by enzymatic digestion for 1 h using a mouse Tumor Dissociation Kit (catalog no. 130-096-730, MACS, Germany) at 37 °C. Digested tissue was passed through a 100 µm cell strainer (catalog no. 352360, Falcon, Corning, USA) and the erythrocytes were selectively lysed with RBC Lysis Buffer Solution (catalog no. R1010, Solarbio Life Sciences, China). Cells were resuspended in Matrigel at 1:1 ratio. 10 µL Matrigel domes were dispensed in 6-well plates (catalog no.150239, Nunc, Thermo Scientific, USA) and 96-well plates (catalog no.11521, Labselect, Labgic, China) and then covered with Cholangiocarcinoma Organoid culture medium (catalog no. K2104-LB, bioGenous, China). The cell culture plate was placed at 37 °C incubator with 5% CO_2_. The culture medium was refreshed every 3–4 days and passaged every 1–2 weeks. Organoids were broken down into small spheres using a 70 μm cell strainer owing to difficult digestion by mechanical dissociation or 0.25% Trypsin-EDTA. They were then seeded onto ultralow-attachment plates at a density of approximately 100 organoids per 100 μL in 5% Matrigel/culture medium and treated with various drug concentrations. Cell viability was assessed after 48 h treatment using a CCK-8 assay.

**Micro-dissected tumor tissues (MDT) model and drug testing**

The microfluidic chip assay was performed as described by Thomas Gervais et al. ^19^. Briefly, the excised tissues from MCA23 subcutaneous tumor and lung metastasis were expeditiously gathered and washed in pre-cooled DMEM medium supplemented with 1% penicillin/streptomycin. After meticulously trimming the tissues were into approximately 1 mm-sized strips using surgical scissors, they were processed with a 500 μm Tissue Punch (catalog no. PUN0500, Zivic Instruments, Pittsburgh, USA) to generate consistently sized tissue fragments. The final product was a cylindrical MDT measuring around 300 μm tall and 380 μm wide. These MDTs were subsequently inserted into pre-sterilized microfluidic chips, with the medium being refreshed every three days. The MDTs were then subjected to treatment with different concentrations of indicated drugs. After a 7-day drug treatment, tissue fragments were isolated, stained with PE-Annexin V and 7-AAD for 15 min at room temperature according to the manufacturer’s instructions (PE Annexin V Apoptosis Detection Kit I, catalog no. 559763, BD, USA), and analyzed by flow cytometry to measure apoptosis rates and tissue response to the drug. For on-chip analysis, dual-fluorescent staining with CellTracker™ Green CMFDA (CTG, catalog no. C7025, Thermo Fisher Scientific, USA) and PI (catalog no. 550825, BD Biosciences, USA) was performed at endpoint time after the indicated drug treatment. CTG labeled viable cells and PI labeled dead cell nucleic acids. HBSS solutions with CTG (5 μM) alone or CTG (5 μM) and PI (1.5 μM) were added sequentially to the systems. MDTs were first incubated with CTG for 1 h and subsequently with both dyes for an additional 30 min. After replacing the dye solutions with hank’s balanced salt solution, the samples were imaged under a microscope (Zeiss Imager. Z2, China).
